# Associations between injury occurrence and environmental temperatures in the Australian and German professional football leagues

**DOI:** 10.1097/EE9.0000000000000364

**Published:** 2025-01-22

**Authors:** Edgar Schwarz, Rob Duffield, Donna Lu, Hugh Fullagar, Karen aus der Fünten, Sabrina Skorski, Tobias Tröß, Abed Hadji, Tim Meyer

**Affiliations:** a Saarland University, Institute of Sports and Preventive Medicine, Campus Geb B8 2, Saarbrücken, Germany; b University of Technology Sydney, Faculty of Health, School of Sport, Exercise and Rehabilitation, Ultimo, NSW, Australia; c University of New South Wales, Kensington, NSW, Australia; d Reykjavik University, Department of Sport Science, Reykjavik, Iceland.

**Keywords:** Epidemiology, Heat, Team sport, Thermoregulation, WBGT

## Abstract

A cross-sectional analysis was performed to investigate associations between environmental temperatures and injury occurrence in two professional male football (soccer) leagues. Data from seven seasons of the German Bundesliga (2142 matches) and four seasons of the Australian A-League (470 matches) were included. Injuries were collated via media reports for the Bundesliga and via team staff reports in the A-League and comprised injury incidence, mechanisms (contact, noncontact), locations (e.g., ankle, knee, and thigh), and types (e.g., muscle and tendon, joint and ligament). Weather data included ambient air temperature (temperature or T) and wet bulb globe temperature (WBGT), which were collected from online sources retrospectively. Generalized linear mixed models were analyzed to examine associations between temperature or WBGT and injury occurrence for each league, respectively. Additionally, matches were grouped into categories of 5°C temperature steps to compare for injury occurrence. Results showed no relationship existed between either temperature or WBGT and any injury occurrence, mechanisms, locations or types for the Bundesliga (*P* > 0.10). A trend for an increase in injury occurrence in higher WBGT existed in the A-League (*P* = 0.05). Comparisons between 5°C temperature categories showed no significant differences for injury occurrence for either temperature or WBGT in either League (*P* > 0.05). Within the observed temperature ranges (−11.2 to 37.1°C T; −12.2 to 29.6°C WBGT) environmental temperature had no relationship with the rate or type of injury occurrence in professional football. Nevertheless, the number of matches at extreme heat within this study was limited and other factors (e.g., playing intensity, season stage, ground conditions) likely co-influence the relationship with injuries.

**What this study adds**

This study is the first to investigate associations between injury occurrence and environmental conditions in association football. Overall, 2101 injuries in 2612 matches were observed across two professional leagues. Of those investigated leagues, one showed no relationship between injury occurrence and environmental temperatures, the other demonstrated a small relationship between more injuries and higher wet bulb globe temperature. Therefore, further investigation, including more observations at higher temperatures, is needed.

## Introduction

Injuries in football (soccer) are common and remain the biggest concern for player health and performance, as well as for team performance.^1,2^ The risk of injury is generally higher in football compared to other workforces, and occupational health and safety strategies need to focus on reducing this risk and explore associated factors.^3^ The risk of injury is generally influenced by both intrinsic (e.g., joint flexibility, previous injury, or muscular fatigue) and extrinsic (e.g., environmental conditions, pitch surface, playing schedule, or fouls) risk factors.^4^ Although environmental conditions have been proposed as an external risk factor for injury,^4^ the relationship between injury occurrence and environmental temperature lacks epidemiological insight in football. Nevertheless, initial concerns have led to the Fédération Internationale de Football Association (FIFA) and other professional leagues to introduce heat policies.^5^

Recently, there are growing concerns around playing football in hot environmental conditions.^6,7^ Exercising in hotter temperatures leads to an earlier onset and higher levels of fatigue,^8^ which in football typically translates to a reduction in running performance.^9^ Alongside physical performance changes, there are expected impairments to cognitive function^10^ and technical match play when football is played in the heat.^11,12^ The earlier onset of fatigue and reduced physical and cognitive performance in higher temperatures are hypothesized to lead to an increased susceptibility to injury.^10,13,14^ In the general public as well as in different occupational fields, this has resulted in an increased number of acute and unintentional injuries in hotter environmental temperatures, although the potential mechanisms of this relationship are still under investigation.^13,15,16^ It was hypothesized that a number of factors associated with hotter conditions, such as changes in cognitive performance, impaired balance, more risky behavior, increased muscle fatigue, dehydration, and poorer sleep quality, could influence the increased injury occurrence.^13,17^ In laboratory studies, hyperthermia-induced fatigue decreased attention and increased errors in complex tasks,^18^ potentially leading to errors in planning and performing movement strategies, such as, tackles or landings in football. Furthermore, there is an impaired coordination, especially proprioception,^19^ dynamic and static postural stability, and balance.^14,20^ For example, a combination of hypohydration and hyperthermia has been shown to lead to more landing and balance errors and higher center-of-pressure and elliptical sways during a dynamic balance test.^14^ Finally, there is also a tendency to take more risky decisions in hotter temperatures.^21^ Despite the hypothesized nature of these mechanisms insights from larger epidemiological investigations on this relationship of environmental temperature with injury in football remain scarce. While football federations and teams are concerned about the effect of environmental conditions on match technical and physical performance,^9,11^ there should also be a concern about the injury risk in such conditions.^6^

The few existing epidemiological studies investigating the relationship between environmental conditions and injuries in football have shown different injury rates in teams located in warmer compared to those in colder regions.^22–24^ A study in Swedish women’s football found a higher prevalence of injuries in colder regions.^22^ In European professional men’s football, a similar overall trend was observed, though anterior cruciate ligament injuries were more frequent in warmer regions.^24^ In contrast, overall injuries were higher in teams based in hotter environments in Australian Rules football.^23^ As these varying results are based on a dichotomous regional categorization, and no actual weather data was recorded, the differences observed could be resulting from different competition structures, differences in training and nutritional habits, different playing styles and levels, different medical support systems, as well as the different climates, or a combination of factors.^22–24^ Due to this methodology, previous studies rarely describe the relationship between environmental temperature and injury rates and further investigation as an extrinsic injury risk factor is required.

Although in the general public, injury occurrence seems to increase with higher environmental temperatures,^13,15,16^ this association was reversed in track and field athletes, especially regarding muscle injuries in sprint disciplines.^25^ In team sports, only two studies in rugby have reported match and training temperatures alongside injury occurrence.^26,27^ Both reported no significant relationships between injuries and the recorded weather conditions, which ranged from ambient air temperature (temperature or “T”) and relative humidity to wind chill temperature. None of these studies has investigated wet bulb globe temperature (WBGT) a “feels-like temperature” combining the influence of temperature and relative humidity but also solar radiation and wind speed, that is often used in sport federations heat policies.^28^ These previous studies in Rugby suggested playing style and match intensity, as well as ground conditions, as potential extrinsic risk factors of greater importance than the environmental conditions.^26,27^ Furthermore, physical demands change in different parts of the playing season, while climatic conditions change according to the seasons. Therefore, suggesting that multiple factors could all influence injury occurrence and more data on environmental and contextual aspects (e.g., stage of the season, congested periods, playing styles, match characteristics) were required to investigate a possible influence on injury risk based on environmental temperature.^26,27^ Accordingly, this study aimed to explore relationships between match environmental temperatures and injury occurrence in football (soccer), by analyzing a large number of observations from two professional leagues from different continents, representing a range of different climates. We hypothesize that injury occurrence increases when match temperature is higher.

## Methods

Data of 2612 matches of male professional football (soccer) were obtained. This included all 2142 matches, over seven seasons (2014–2021) of the German “Bundesliga” (BL), and 470 of 624 matches, over four seasons (2016–2020) of the Australian “A-League” (AL). In the AL, one team was located outside Australia, in Wellington, New Zealand. The BL dataset was built on open-source data used in previous research.^29,30^ It involved scouting multiple online and social media sources to detect and describe injuries. The injury definition for the BL data was based on Fuller et al:^31^ when a player could not fully participate in at least 1 day of training or competition. If a player had to stop exercise because of injury but returned the next day, this counted as a time loss of zero days and was not included, as these types of injury were insufficiently reported by media and clubs.^29,30^ The AL data set was based on anonymized data used in previous research.^32^ In the AL, injuries were defined as “any physical complaint requiring medical attention resulting in a missed AL match” (also adopted from Fuller et al)^31^ and had been obtained based on injury surveillance system within the league, where injury data was collected from team medical staff on a weekly basis. Due to the different data sources, leagues were analyzed separately, and no grouped analysis was performed, though using two available data sources from different leagues and continents provides larger and wider insights on the influence of temperature on injury occurrence. Ethical approval was granted by the Ethics Committee of the Faculty for Human and Business Sciences of Saarland University (Ref No.: 23-14).

Injuries for both leagues were further classified by mechanism (contact versus noncontact), where contact injuries resulted from physical contact with another player or object, and noncontact injuries occurred without any interaction with outside forces.^33^ Injury locations and types were classified according to the Orchard Sports Injury Classification System (OSICS).^31^ Grouping the head and neck, upper limbs, and trunk injuries, and adding heat illnesses, this resulted in nine distinct injury locations (head and neck, upper limbs, trunk, hip/groin, thigh, knee, lower leg/Achilles tendon, ankle, foot/toe), six different injury types (muscle and tendon, joint [nonbone] and ligament, contusions, fractures and bone stress, lacerations and skin lesions, central/peripheral nervous system), as well as heat illnesses. Injuries were aggregated to overall counts and by subgroups (mechanism, location, and type) across both leagues for each match and reported as injuries per match. Although reporting injuries per 1000 match-hours is the more common , the data was reported as injuries occurring per match per team, due to the lack of specific player exposure data. The limitations associated with this method should be recognized.^34^

Environmental conditions in the form of temperature and WBGT were collated retrospectively for each match. The use, advantages, and disadvantages of WBGT have been described extensively in previous research.^28,35,36^ Despite its widespread use, the black globe temperature (radiative heat gain) and natural wet bulb temperature (evaporative heat loss) measurements are criticized as not representing human thermoregulation, thereby underestimating heat stress in many settings.^28,37^ It should also be mentioned that WBGT is a heat stress index and is not validated for colder conditions. Therefore, to interpret the effects of colder environments on injury occurrence, temperature was also used in our analyses. Although more modern and sophisticated thermal indexes exist,^37,38^ WBGT remains widely used, especially in sports federation heat policies. Specifically, this index is also used in the heat policy introduced by FIFA, which recommends the use of drinking breaks at 32°C WBGT.^5^

For BL matches, weather data were obtained from Meteostat.net.^39^ This is an open-source service, providing hourly meteorological data for any given coordinates. Data is obtained as a weighted interpolation depending on the distance and elevation difference from the four closest weather stations to a geological location. They provide the following data: temperature, relative humidity, dew point, wind speed, air pressure, total precipitation, and the current weather condition. Based on this, WBGT can be estimated in a variety of ways according to previous research.^35^ We used the estimation developed by Liljegren et al.^36^ This is validated and reliable in different environmental settings and is described as the best estimate for WBGT from different methods.^40^ The R code needed to implement these calculations has been provided and used in previous research.^41^ Wind speed was assumed to be a minimum of 1 m/s, as moving players generate airflow of at least equivalent to that. Solar radiation was estimated using the solar angle at the time and location of the match.^42^ As Meteostat.net provides hourly data, two time points (the kick-off time and one hour later) were used per match and averaged. If the match did not start at a full hour but at 15 or 30 minutes past the hour, the previous full hour was used as a starting point and the following hour as a second time point. For AL matches, environmental conditions were provided by UBIMET.com.^43^ This commercial provider uses artificial intelligence and data input from multiple weather stations, radar, and satellite data to estimate meteorological data at given ground locations. They provide temperature, relative humidity, solar radiation, and WBGT measurements for the starting times of the first and second half, which were then averaged to create one value per match. To validate the WBGT data based on Meteostat.net data, the WBGT estimation method used for the BL data was also performed with the AL data. As internal validation, results were then compared to the WBGT reported from UBIMET.com. There was a very good linear association (correlation coefficient r = 0.93).

Both temperature and WBGT were used for the analysis. They were treated as continuous variables to investigate relationships between environmental conditions and injury occurrence. Additionally, matches were categorized into groups of 5°C temperature increments, resulting in nine WBGT groups (from −15 to 30°C) and 10 temperature groups (−15 to 35°C) in the BL and five WBGT groups (5–30°C) and six temperature groups (5–35°C) in the AL.

Statistical analyses were performed separately for each league due to the different injury definitions and data sources (media collation vs. team reports) used. Any over- or underreporting of injuries by one given reporting method would influence all matches regardless of the environmental conditions and therefore not alter the relationship between temperature and injuries within a league. Further, separate analyses do not conflate acclimatization and local exposures of the players given the different temperature ranges regularly encountered by players in their respective leagues. To analyze the relationship of environmental conditions and injury, generalized linear mixed effects models (GLMM) were used, with specified zero-inflated Poisson distribution of the injury data and a random effect of the observed match. Additionally, for the separate temperature categories in 5°C steps, differences between categories were determined by one-way analysis of variance (ANOVA) with Tukey’s Honest Significant Difference post-hoc test. Analysis was performed with *R Studio 2022.07.1* using *R version 4.2.1 stats*, *jtools,* glmmTMB, *MuMIn* & *performance* libraries. Statistical significance was defined at a level of 5% or less for the α-error (*P* < 0.05).

## Results

As outlined in Table 1, 2101 injuries (BL: n = 1779, AL: n = 322) were reported from 2612 matches, resulting in 0.83 ± 0.97 injuries per match in the BL and 0.68 ± 0.86 injuries per match in the AL. Contact (BL: n = 881 [49.5%]; AL: n = 148 [46.1%]) and noncontact injuries (BL: n = 898 [50.5%]; AL: n = 174 [54.2%]), are similarly distributed within both leagues. The lower limbs are the most common injury location (BL: n = 1417 [79.7%]; AL: n = 264 [82.2%]). Muscle and tendon injuries are the most frequent injury type (BL: n = 855 [48.1%]; AL: n = 140 [43.6%]). Data on specific injury mechanisms, locations and types per match are reported in Table 1 for each league separately. Table 1 also shows the environmental conditions for both leagues. Although maximum values are similar, a larger proportion of the BL matches is played in colder conditions. The number of matches above thresholds implemented by respective governing bodies is minimal in both leagues, with 27 matches (11 in BL, 16 in AL) above 26°C WBGT or 31°C T, two matches (zero in BL, two in AL) above 28°C WBGT and none above 32°C WBGT).

**Table 1.** Descriptive summary of environmental conditions and injury occurrences per match in 4 seasons of A-League and 7 seasons of Bundesliga

Variable	A-League	Bundesliga
Number of matches Number of seasons	470 4	2142 7
Mean temperature (^o^C)	21.0 ± 5.3	11.0 ± 7.2
Temperature range (°C)	7.6–37.1 (∆29.5)	−11.2 to 34.3 (∆45.5)
Mean WBGT (°C)	18.1 ± 4.2	9.4 ± 6.0
WBGT range (°C)	6.2–29.6 (∆23.4)	−12.2 to 26.3 (∆38.5)
Total number of injuries	322	1779
Number of injuries per match Noncontact Contact Muscle and tendon Joint (nonbone) and ligament Contusion Fractures and bone stress Laceration and skin lesion Central/peripheral nervous system Heat illness Head and neck Upper limbs Trunk Hip/groin Thigh Knee Lower leg/achilles tendon Ankle Foot/toe Lower Limbs (overall)	0.68 ± 0.86 0.36 ± 0.61 0.32 ± 0.58 0.29 ± 0.54 0.16 ± 0.43 0.04 ± 0.20 0.04 ± 0.21 0.00 ± 0.07 0.06 ± 0.28 0.01 ± 0.08 0.05 ± 0.25 0.02 ± 0.16 0.04 ± 0.20 0.06 ± 0.25 0.22 ± 0.47 0.12 ± 0.35 0.07 ± 0.25 0.06 ± 0.28 0.03 ± 0.18 0.57 ± 0.79	0.83 ± 0.97 0.42 ± 0.68 0.41 ± 0.69 0.38 ± 0.67 0.16 ± 0.40 0.15 ± 0.40 0.04 ± 0.21 0.01 ± 0.11 0.04 ± 0.21 0.00 ± 0.00 0.06 ± 0.26 0.03 ± 0.17 0.05 ± 0.23 0.08 ± 0.28 0.21 ± 0.48 0.11 ± 0.34 0.08 ± 0.28 0.12 ± 0.37 0.05 ± 0.23 0.66 ± 0.87

### Bundesliga

**Figure 1.**
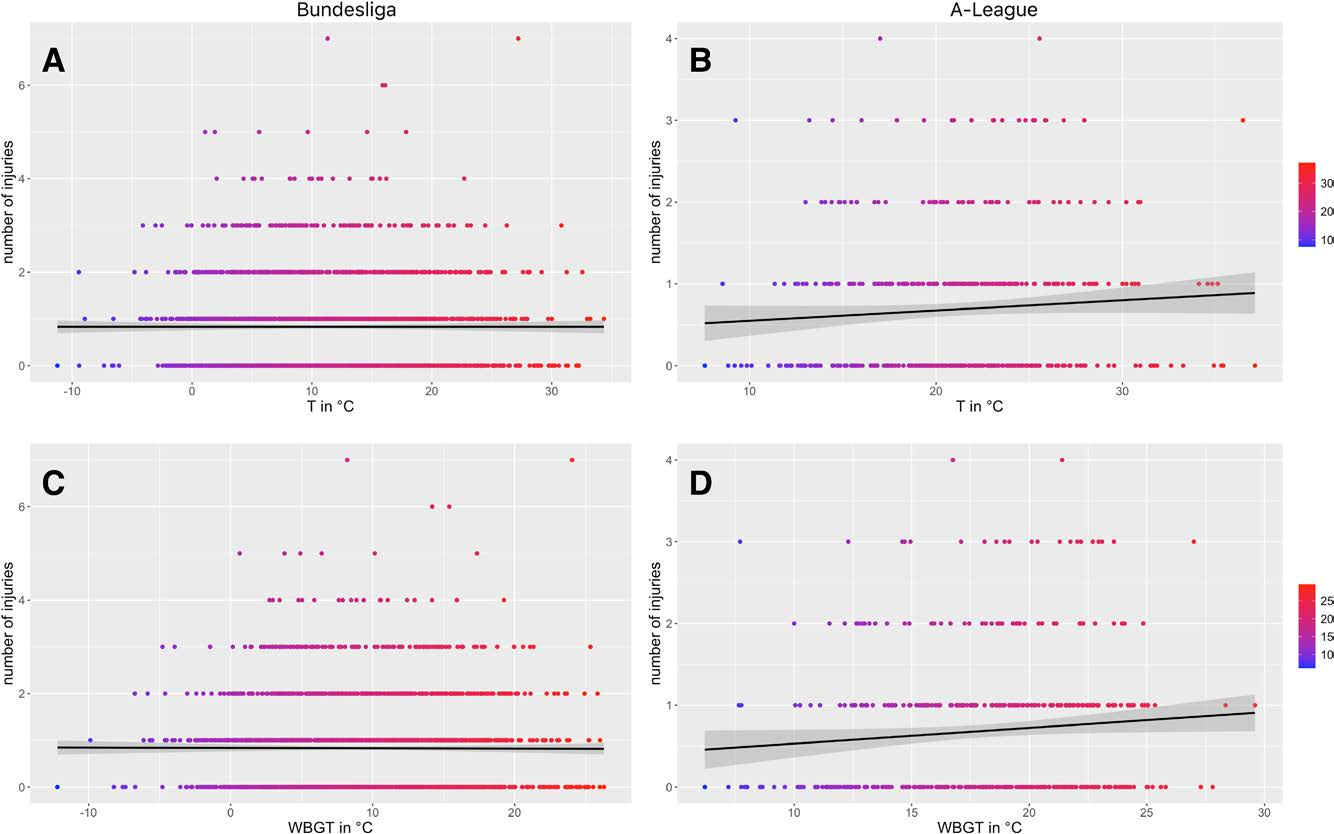
Results of generalized linear mixed models between overall injuries in each league, wet bulb globe temperature (WBGT) and temperature (T). Each dot represents one match, while the black line shows the regression line, with the 95% CI (gray band); A, relationship between injuries and temperature in the Bundesliga. B, relationship between injuries and temperature in the A-League. C, relationship between injuries and WBGT in the Bundesliga. D, relationship between injuries and WBGT in the A-League.

**Figure 2.**
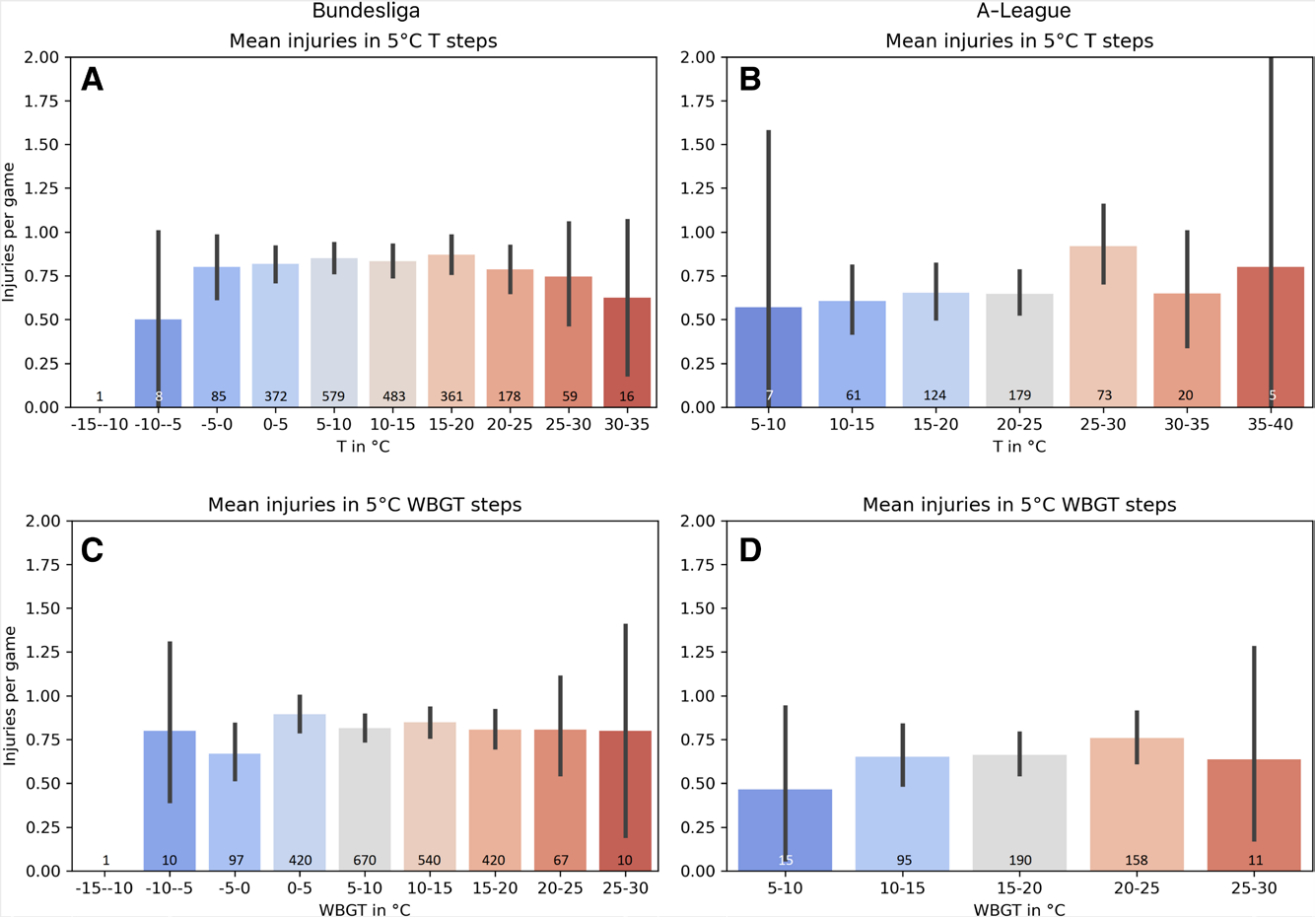
Mean injury-occurrence per match (gray bar) and 95% confidence interval (black line) in different temperature (T) and wet bulb globe temperature (WBGT) zones (The small number on the bars indicating the number of observed matches in each category. A, injuries in 5°C temperature steps in the Bundesliga. B, injuries in 5°C temperature steps in the A-League. C, injuries in 5°C WBGT steps in the Bundesliga. D, injuries in 5°C WBGT steps in the A-League).

In BL there is no significant relationship between temperature or WBGT and injury occurrence (Figure 1; *P* > 0.83). Table 2 shows the GLMMs using temperature or WBGT to predict injury occurrence. There are only marginal differences in the outcomes of using temperature and WBGT. Second, the ‘null-hypothesis model’ (i.e., injuries occur randomly) performs similar, indicating that the random model is equally successful in predicting injuries than models using temperature or WBGT as predictors. When reported based on injury subcategories of mechanism, type, or location, no significant relationship (*P* ≥ 0.10) is evident for any GLMM with either temperature or WBGT. Results of the GLMMs for overall injuries and 5^o^C subcategories are presented in Table 3, with no significant differences (*P* ≥ 0.37) in overall injury occurrence or any injury subcategories existing for either of the temperature or WBGT (Figure 2). As the trends of the subcategories followed those of the overall injuries, their results are not specifically displayed or visualized here. Of note, no heat illness events are reported in the data set.

**Table 2.** Performance of the generalized linear mixed effects models (GLMMs), comparing the “Null”-model to models using temperature (T) or wet bulb globe temperature (WBGT) as independent factor to predict overall injury occurrence

	Coeff	AIC	BIC	Deviance	RMSE	LL	marg. R^2^	*P* value
Bundesliga								
Inj ~ 1 + (1 Match)		5230.4	5247.4	5224.4	0.83	−2612.2		
Inj ~ T + (1 Match)	0.00	5232.4	5255.0	5224.4	0.83	−2612.2	0.00	0.97
Inj ~ WBGT + (1 Match)	0.00	5232.3	5255.0	5224.3	0.83	−2612.2	0.00	0.81
A-League								
Inj ~ 1 + (1 Match)		1044.3	1056.8	1038.3	0.76	−519.2		
Inj ~ T + (1 Match)	0.02	1043.8	1060.4	1035.8	0.76	−517.9	0.008	0.11
Inj ~ WBGT + (1 Match)	0.03	1042.4	1059.0	1034.4	0.76	−517.2	0.009	0.05

**Table 3.** Association between injuries (overall and selected subcategories) and environmental conditions, showing the mean and standard deviation (Mean ± SD), as well as the estimate and 95% confidence interval (Estimate (95%CI)

	Mean ± SD	Temperature				Wet-bulb globe temperature			
		Estimate (95% CI)	β (95% CI)	R^2^ (marg.)	*P* value	Estimate (95% CI)	β (95% CI)	R^2^ (marg.)	*P* value
Overall injuries									
Overall									
BL	0.83 ± 0.97	−0.00 (−0.01, 0.01)	−0.00 (−0.05, 0.05)	0.00	0.98	−0.00 (−0.01, 0.01)	−0.00 (−0.06, 0.04)	0.00	0.81
AL	0.68 ± 0.87	0.02 (−0.00, 0.04)	0.10 (−0.02, 0.21)	0.01	0.11	0.03 (0.00, 0.06)	0.12 (0.00, 0.24)	0.01	0.05
Mechanism									
Contact									
BL	0.41 ± 0.69	0.01 (−0.01, 0.02)	0.04 (−0.04, 0.11)	0.00	0.35	0.00 (−0.01, 0.02)	0.02 (−0.05, 0.10)	0.00	0.55
AL	0.32 ± 0.58	0.01 (−0.02, 0.05)	0.07 (−0.10, 0.24)	0.00	0.44	0.02 (−0.02, 0.06)	0.09 (−0.08, 0.26)	0.00	0.32
Noncontact									
BL	0.42 ± 0.68	−0.01 (−0.02, 0.01)	−0.04 (−0.11, 0.03)	0.00	0.29	−0.01 (−0.02, 0.01)	−0.04 (−0.11, 0.04)	0.00	0.32
AL	0.36 ± 0.61	0.02 (−0.01, 0.05)	0.10 (−0.06, 0.26)	0.00	0.23	0.03 (−0.01, 0.07)	0.13 (−0.04, 0.29)	0.01	0.13
Type of injury									
Muscle and tendon									
BL	0.40 ± 0.67	−0.01 (−0.02, 0.00)	−0.05 (−0.12, 0.03)	0.00	0.21	−0.01 (−0.02, 0.00)	−0.05 (−0.12, 0.03)	0.00	0.23
AL	0.29 ± 0.54	0.01 (−0.02, 0.05)	0.07 (−0.09, 0.24)	0.00	0.39	0.02 (−0.02, 0.06)	0.09 (−0.08, 0.26)	0.00	0.30
Joint (non-bone) and ligament									
BL	0.16 ± 0.40	0.01 (−0.01, 0.02)	0.06 (−0.05, 0.16)	0.00	0.30	0.01 (−0.01, 0.03)	0.05 (−0.05, 0.16)	0.00	0.30
AL	0.16 ± 0.43	−0.01 (−0.06, 0.03)	−0.06 (−0.31, 0.18)	0.00	0.61	0.00 (−0.05, 0.06)	0.01 (−0.23, 0.26)	0.00	0.92
Location of injury (lower limbs)									
Lower limbs overall									
BL	0.66 ± 0.87	0.00 (−0.01, 0.01)	0.00 (−0.06, 0.06)	0.00	0.96	−0.00 (−0.01, 0.01)	0.00 (−0.06, 0.05)	0.00	0.84
AL	0.57 ± 0.79	0.02 (−0.01, 0.04)	0.10 (−0.03, 0.23)	0.00	0.15	0.03 (−0.00, 0.06)	0.13 (−0.00, 0.27)	0.01	0.06
Hip/groin									
BL	0.08 ± 0.28	0.01 (−0.03, 0.04)	0.04 (−0.21, 0.30)	0.00	0.73	0.01 (−0.03, 0.05)	0.05 (−0.21, 0.31)	0.00	0.70
AL	0.06 ± 0.25	0.03 (−0.10, 0.16)	0.15 (−0.55, 0.85)	0.00	0.67	0.04 (−0.13, 0.21)	0.16 (−0.56, 0.88)	0.00	0.66
Thigh									
BL	0.21 ± 0.48	−0.01 (−0.02, 0.00)	−0.08 (−0.19, 0.02)	0.00	0.13	−0.01 (−0.03, 0.00)	−0.09 (−0.19, 0.02)	0.00	0.10
AL	0.22 ± 0.47	0.03 (−0.01, 0.07)	0.15 (−0.05, 0.36)	0.01	0.14	0.05 (−0.00, 0.10)	0.20 (−0.01, 0.42)	0.01	0.06
Knee									
BL	0.11 ± 0.34	0.01 (−0.01, 0.03)	0.08 (−0.08, 0.24)	0.00	0.32	0.01 (−0.01, 0.03)	0.07 (−0.09, 0.23)	0.00	0.38
AL	0.12 ± 0.35	−0.01 (−0.07, 0.06)	−0.04 (−0.36, 0.29)	0.00	0.83	0.02 (−0.06, 0.09)	0.07 (−0.25, 0.40)	0.00	0.65
Lower leg/achilles tendon									
BL	0.08 ± 0.28	−0.01 (−0.04, 0.02)	−0.07 (−0.27, 0.13)	0.00	0.30	−0.01 (−0.05, 0.02)	−0.08 (−0.28, 0.12)	0.00	0.46
AL	0.07 ± 0.25	0.03 (−0.04, 0.09)	0.14 (−0.21, 0.48)	0.00	0.44	0.04 (−0.11, 0.19)	0.17 (−0.47, 0.82)	0.00	0.60
Ankle									
BL	0.07 ± 0.28	0.01 (−0.03, 0.05)	0.07 (−0.21, 0.35)	0.00	0.64	0.01 (−0.04, 0.06)	0.07 (−0.21, 0.35)	0.00	0.64
AL	0.06 ± 0.28	0.02 (−0.13, 0.17)	0.09 (−0.70, 0.89)	0.00	0.82	0.02 (−0.18, 0.20)	0.06 (−0.75, 0.87)	0.00	0.88
Foot/toe									
BL	0.05 ± 0.23	0.03 (−0.02, 0.08)	0.22 (−0.14, 0.59)	0.00	0.23	0.03 (−0.03, 0.09)	0.20 (−0.17, 0.57)	0.00	0.30
AL	0.03 ± 0.18	−0.01 (−0.22, 0.20)	−0.06 (−1.18, 1.05)	0.00	0.91	−0.03 (−0.29, 0.23)	−0.14 (−1.24, 0.96)	0.00	0.80

Standardized estimate and 95% confidence interval (β [95% CI]), the marginal R^2^ (R^2^ [marg.]) and *P* value for generalized linear mixed models in the Bundesliga (BL) and A-League (AL).

### A-League

**Figure 3.**
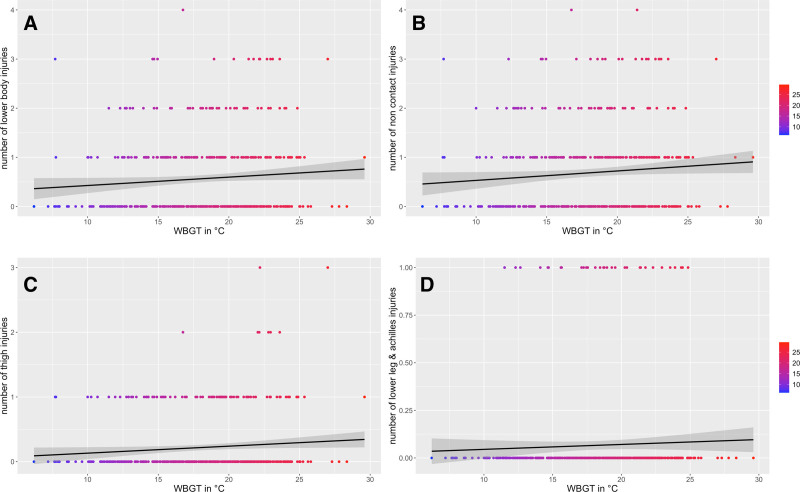
Results of generalized linear mixed models between wet bulb globe temperature (WBGT) specific injury subcategories in the A-League. Each dot represents one match, while the black line shows the regression line, with the 95% CI (gray band). A, overall lower body injuries. B, noncontact injuries. C, thigh injuries. D, lower leg and achilles tendon injuries.

In the AL there are 0.03 (95 CI = 0.00, 0.06) more injuries per 1°C WBGT increase, though it is only describing a small effect (estimated coefficient [β] = 0.12, 95 CI = 0.00, 0.24; *P* = 0.05) and a neglectable amount of explained variance (R^2^ = 0.01). This association is not significant and lower when temperature is used as a predictor for injuries, which then increases by 0.02 per 1°C temperature increase (95 CI = 0.00, 0.04; R^2^ = 0.01; β = 0.10, 95 CI = −0.02, 0.21; *P* = 0.11). A similar tendency toward more injuries in higher WBGT is observed for all lower body injuries combined (0.03; 95 CI = 0.00, 0.06; R^2^ = 0.01; β = 0.13, 95 CI = −0.00, 0.27; *P* = 0.06) and thigh injuries specifically (0.05; 95 CI = 0.00, 0.10; R^2^= 0.01; β = 0.20, 95 CI = −0.01, 0.42; *P* = 0.06). Further, small effect sizes are observed for noncontact (β = 0.13), hip and groin (β = 0.16), and injuries of the lower leg and Achilles tendon (β = 0.17) that occur more often when WBGT is higher, but none of these effects are significant (*P* > 0.05; Figure 3). Similar findings exist for temperature in the models, with GLMM outcomes for overall injuries and selected subcategories reported in Table 3. Finally, there are three heat illnesses in the data set, occurring at 22°C, 25°C, and 29°C WBGT.

## Discussion

This study was the first to investigate the relationship of environmental match temperatures (T and WBGT) on injury occurrence in professional football. Regardless of whether temperature or WBGT was considered, environmental temperatures were largely unrelated to the overall number of injuries, injury mechanism, location or type; however, there was a tendency toward a small effect of more injuries occurring in higher WBGT in the AL. When comparing matches in classification of 5°C temperature or WBGT increments there were no differences between any of the injury subcategories in either league.

In the AL, the influence of WBGT on the overall injury occurrence tended towards significance (*P* = 0.05), indicating that when WBGT increased by 1°C, there was an increase of 0.03 injuries per match (for all 22 players combined). However, the effect size and explained variance were minimal, leading to an inconclusive interpretation of this relationship. In the BL there were no associations between either temperature or WBGT and injury outcome variables. The lack of a clear relationship between hot environmental temperatures (temperature or WBGT) and increased injuries contrasts with our hypothesis of an increased injury rate due to premature fatigue and impairments in, coordinative and cognitive function in hotter temperatures.^10,20,44^ It further contrasts with research from occupational industries that reported an increase of acute injuries in industrialized settings when workers are exposed to hotter conditions.^13^ Although the potential mechanisms of this relationship are still under investigation, this was hypothesized to be the result of changes in cognitive performance, impaired balance, more risky behavior, increased muscle fatigue, dehydration, and poorer sleep quality.^13^ In the specific setting of outdoor team sports, this association is yet to be confirmed. As previous studies in rugby suggest, sport-specific factors might mitigate or mask the effects of heat exposure, that are found in other settings. These could include adjustments of playing intensity and style in the heat, influence of weather on ground conditions, and systematically differing demands in various parts of the football season.^26,27^ For example, it has been shown that players decrease running distances and playing intensity when playing in hotter environments, reducing their risk of hyperthermia and thermal fatigue.^9,45^ Nevertheless, it has also been shown, that high core temperatures can be reached in football players despite reductions in running distances, but also without noticeable health adversities.^46^ By decreasing playing intensities in hotter conditions, the effects of higher heat stress might be mitigated, reducing the effects on cognitive and coordinative capacities shown in laboratory or occupational settings. Lower playing intensities have further been shown to lead to less injuries, for example, when comparing training to matches.^1,29^ Additionally, higher temperatures and humidity lead to softer ground conditions, which have been linked to a lower injury occurrence,^26^ possibly counteracting an increase in injuries caused by the effects of heat.

The environmental conditions in these two leagues represent a wide range of conditions that may also be observed in many other settings around the world. Nevertheless, some competitions will be held in hotter conditions due to their scheduling in the hotter summer months (e.g., national tournaments, including youth competitions, European competition qualification matches, and preseason matches). In the BL, the exposure to heat remained rare due to the off-season period over the summer and mostly cold-to-mild climates during playing months. There were no matches at extreme risk (>27.9°C WBGT), four at high risk (25.7–27.8°C WBGT) and 40 at moderate risk (22.3–25.6°C) for heat-illnesses according to American College of Sports Medicine guidelines.^47^ In the AL, the overall distribution of matches indicated hotter temperatures compared to the BL, which is understandable given that in Australia, in general warmer temperatures are recorded compared to Germany and it is a summer-based competition. However, the scheduling of many matches after 5pm may explain the reduced exposure to extreme environmental temperatures.^48^ There were four matches kicked off at extreme risk(>27.9°C WBGT), five at high risk (25.7–27.8°C WBGT) and 84 at moderate risk (22.3–25.6°C) for heat illnesses according to American College of Sports Medicine categories.^47^ In line with the relevant governing body heat policy guidelines, 16 matches contained an additional short drinking break in each half when WBGT was higher than 26°C or temperature was above 31°C. Previous research proposed that for practical topics related to exercising in the heat, using WBGT may not be advantageous over using temperature.^28^ The current data also revealed only small differences in the models using temperature or WBGT. However, it needs to be considered that WBGT was estimated based on data from weather stations and a WBGT measurement on the field of play may be more advantageous compared to obtaining only temperature. Nevertheless, given the additional difficulties in measuring WBGT (e.g., need for expensive equipment and trained staff), the consideration of appropriate environmental measures to enact heat policies or interventions remains for ongoing research and discussion. Especially in leagues lacking resources, the use of WBGT might not be applicable. Furthermore, previous publications pointed toward limitations of WBGT in describing high humidity climates, underestimating the actual heat stress posed onto players.^49^ Of note, two of the three observed heat illnesses in the current study were reported in a moderate WBGT of ~22°C and temperature of ~25°C, but high relative humidity of more than 65%.

Despite this novel investigation, some limitations require consideration when interpreting the findings. First, the retrospective approach to capture environmental conditions was the best available method for this study. An estimation of the WBGT based on basic meteorological data,^36^ with assumptions for solar radiation and wind speed, was used. Although this method has been shown to give reliable estimates,^35^ on-site measurements would have been preferable and should be encouraged in the future. Especially solar radiation and air flow may differ locally inside football stadiums and their accuracy would benefit most from a measurement at the athletes field of play. Another limitation involves the collation of injury data from different leagues, using different data sources, which prevents the comparison between or the collation of BL and AL data. However, any over- or underreporting of injuries by one given method would be influencing all matches, regardless of the environmental conditions, therefore also not altering the investigated association. To conclude, the retrospective approach to collate weather data and inclusion of different leagues allowed for the investigation of a large amount of observed match data, accepting that this resulted in a less standardized data collection. Another limitation was the low number of matches in extreme heat conditions, as well as an inability to include variables such as acclimatization, stage of the season, prior exposure etc., which were not available. Therefore, further investigations of more extreme conditions may be warranted to account for further confounding factors.

In conclusion, there was no significant relationship between environmental temperatures and injury occurrence in male professional football matches within the observed temperature range. This relationship may be co-influenced by altered playing intensities and ground conditions in changing temperatures. Both leagues had a low exposure to challenging hot environmental conditions, likely due to scheduling matches outside the hottest periods of the year or day. With increasing global temperatures, this strategy may be applied by football governing bodies, to ensure the occupational health and safety of football players, as the effects of matches in hotter conditions, multiple consecutive heat exposures or abrupt changes from cool to hot environments on injury occurrence remain to be investigated.

## Conflicts of interest statement

The present results do not constitute endorsement by either of the two involved leagues. The results of the study are presented clearly, honestly, and without fabrication, falsification, or inappropriate data manipulation. E.S. receives a scholarship from the “Deutsche Fußball Liga GmbH” (DFL) that is operating the German BL. T.M. is head of a DFL working group entitled “Medicine in Professional Football” and chairman of the medical committees of the German FA (DFB) and the European football confederation (UEFA). R.D. is Head of Research & Development at Football Australia. D.L. is the Injury Surveillance Officer at Football Australia. There were no further conflicts of interest. Other authors declare that they have no conflicts of interest with regard to the content of this report.

## ACKNOWLEDGMENTS

The authors thank Football Australia for sharing the A-League injury reports and the DFL for funding the PhD scholarship of ES.

## Supplementary Material


