# Supplementary material for: Associations between injury occurrence and environmental temperatures in the Australian and German professional football leagues
Source: Environ Epidemiol. 2025 Jan 22;9(1):e364. doi: 10.1097/EE9.0000000000000364 (PMC11756877; doi:10.1097/EE9.0000000000000364)
Supplement: Supplementary file 1 [file ee9-9-e364-s001.pdf]

Supplementary code 1: Example code to generation of generalized linear models in R:

```
#relationship between WBGT (W) and Injury (I)
```

Generalized linear model with specific poisson distribution of data:

Model

```
#generalized linear model (glm)
library(stats)

#glm poisson
mod1 <- glm(I ~ W,family = 'poisson', data =dfBL)
```

Null Model:

```
#glm poisson NULL
mod0 <- glm(I ~ 1,family = 'poisson', data =dfBL)
```

Generalized linear model with specific zero inflated poisson distribution of data:

Model

```
#zero inflated glm poisson (zeroinfl) or (glmmTMB)
library(pscl)

modzi1 <- zeroinfl(I~W | 1, data=dfBL, dist="poisson")

#zero inlated glm poisson NULL
modzi0 <- zeroinfl(I~1 | 1, data=dfBL, dist="poisson")
```

OR:

```
modzi1 <- glmmTMB(I ~ W, zi = ~ 1, data = dfBL,family = poisson)
modzi0 <- glmmTMB(I ~ 1, zi = ~ 1, data = dfBL,family = poisson)
```

Generalized linear mixed-effects model with specific poisson distribution of data:

Model

```
#generalized linear mixed-effects model (glmm)
library(lme4)

#glmm poisson
mmod1 <- glmer(I ~ W + (1|ID_E), family = 'poisson', data=dfAL)

#glmm poisson NULL
mmod0 <- glmer(I ~ 1 + (1|ID_E), family = 'poisson', data=dfAL)
```

Generalized linear mixed-effects model with specific poisson distribution of data:

Model

```
#zero inflated glmm poisson
library(glmmTMB)

mmodzi1 <- glmmTMB(I ~ W  + (1|ID_E), zi = ~ 1, data = dfAL,family = poisson)

#zero inflated glmm poisson NULL
mmodzi0 <- glmmTMB(I ~ 1  + (1|ID_E), zi = ~ 1, data = dfAL,family = poisson)
```

Supplementary code 2: Example code to interpret performance of generalized linear models in R:

```
Generalized (Mixed) Linear Model Performance Measurements:
#RMSE:
sqrt(mean(residuals(MODEL)^2))

#R2
library(MuMIn)
r.squaredGLMM(MODEL)

#AIC
AIC(MODEL)

#BIC
BIC(MODEL)

#overall
library(performance)
check_model(MODEL)

summary(MODEL)

qqnorm(resid(MODEL))+qqline(resid(MODEL))

model_performance(MODEL)

anova(MODEL1, MODEL0)
```

Supplementary code 3: Example code to analyse difference between temperature categories (heatw) in Python:

Generation of heat categories of 5 °C WBGT steps:

```
df["heatw"] = pd.cut(
      x=df["WBGT C"],
      bins=[-15, -10, -5, 0, 5, 10, 15, 20, 25, 30, 35],
      labels=["-15--10", "-10--5", "-5-0", "0-5", "5-10", "10-15", "15-20", "20-25", "25-
      30"],)
```

ANOVA to compare categories

```
import pingouin as pg

aov = pg.anova(data=df, dv='Zweikämpfe', between="heatw", detailed=True)

print(aov)
```

Post Hoc Tests

```
pt = pg.pairwise_tukey(dv='F', between='heatw', data=df)

print(pt)

comparison = MultiComparison(df["F"], df["heatw"])

comparison_results = comparison.tukeyhsd()

print(comparison_results.summary())
```
